# Dissecting the molecular trajectory of fibroblast reprogramming to chemically induced mammary epithelial cells

**DOI:** 10.3389/fcell.2023.1194070

**Published:** 2023-08-02

**Authors:** Liangshan Qin, Dandan Zhang, Siyi Liu, Quanhui Liu, Mingxing Liu, Ben Huang

**Affiliations:** ^1^ College of Animal Science and Technology, Guangxi University, Nanning, China; ^2^ Guangxi Academy of Medical Sciences, Nanning, China

**Keywords:** reprogramming, small-molecule compounds, induced mammary epithelial cells, molecular trajectory, transdifferentiation

## Abstract

**Introduction:** The plasticity of cell identity allows cellular reprogramming that manipulates the lineage of cells to generate the target cell types, bringing new avenues for disease modeling and autologous tailored cell therapy. Previously, we had already successfully established a technical platform for inducing fibroblast reprogramming to chemically induced mammary epithelial cells (CiMECs) by small-molecule compounds. However, exactly how the molecular mechanism driving the lineage conversion remains unknown.

**Methods:** We employ the RNA-sequencing technology to investigate the transcriptome event during the reprogramming process and reveal the molecular mechanisms for the fate acquisition of mammary lineage.

**Results:** The multi-step reprogramming process first overcomes multiple barriers, including the inhibition of mesenchymal characteristics, pro-inflammatory and cell death signals, and then enters an intermediate plastic state. Subsequently, the hormone and mammary development genes were rapidly activated, leading to the acquisition of the mammary program together with upregulation of the milk protein synthesis signal. Moreover, the gene network analyses reveal the potential relationship between the TGF-β signaling pathway to mammary lineage activation, and the changes in the expression of these genes may play important roles in coordinating the reprogramming process.

**Conclusion:** Together, these findings provide critical insights into the molecular route and mechanism triggered by small-molecule compounds that induce fibroblast reprogramming into the fate of mammary epithelial cells, and they also laid a foundation for the subsequent research on the development and differentiation of mammary epithelial cells and lactation.

## Background

During the development of organisms, cell fate is determined by a complex set of transcription factors and an epigenetically programmed process that control the differentiation, which are considered irreversible for a long time (Meir et al., 2021). However, the revolutionizing somatic cell reprogramming/transdifferentiation technologies has broken the traditional notions and become one of the research hotspots in the field of life science and regenerative medicine (Hybiak et al., 2020). This is a method of using external factor inductions to break the original gene expression pattern of somatic cells and establishing a new one, so as to reverse the terminally differentiated cell into a multipotent cell or various functional cells (Vierbuchen et al., 2012). The methods of somatic cell nuclear transfer, transfection of specific transcription factors, cell fusion, and induction of small-molecule compounds were now available to the generation of reprogramming cells (Cieślar-Pobuda et al., 2017). Small-molecule compounds can efficiently and reversibly regulate target proteins, so the biological effects are typically rapid (Li et al., 2013). In addition, small-molecule compounds were dose dependent, without foreign gene interventions, and avoid safety concerns that have potentials for therapeutic application (Xu et al., 2015). Moreover, the small-molecule compounds have been widely used to improve reprogramming/transdifferentiation by acting on signaling pathways, epigenetic modifications, and metabolic processes (Qin et al., 2017). To date, a variety of cell types, including iPSCs (Guan et al., 2022), functional neurons (Cheng et al., 2015; Ma et al., 2019), cardiomyocytes (Cao et al., 2016), and pancreas *β* cell (Fomina-Yadlin et al., 2010), were able to be generated by small-molecule compound induction methods from somatic cells.

Reprogramming is a gradual process: the small-molecule compounds trigger widespread disturbance of transcriptome levels and gradual loss of cellular identity and a concomitant activation of the new cellular regulatory network. The mechanism of coordinated changes in cellular plasticity and identity is critical for the reprogramming process (Huyghe et al., 2022). Although the description of reprogramming roadmaps of reprogramming cells has been reported and most of them were about how to activate and maintain the pluripotent network (Chronis et al., 2017; Polo et al., 2012; Stadtfeld et al., 2008), the molecular mechanisms coordinating the stepwise gain of plasticity and the conversion of identity remain largely unknown in direct lineage reprogramming.

Previously, we have demonstrated that efficient goat ear fibroblasts (GEFs) reprogrammed into chemically induced mammary epithelial cells (CiMECs) with lactation function can be accomplished by treatment with a cocktail of five small molecules [BFRTV, including TTNPB (B), forskolin (F), RepSox (R), tranylcypromine (T), and VPA (V)] (Zhang et al., 2021). However, the molecular mechanisms and the lineage conversion process underlying BFRTV induction were not well understood, which limits the strategy optimization to increase the reprogramming efficiency. In this study, we used the bulk transcriptome and single-cell transcriptome sequencing technology to analyze the transcriptome dynamics during the reprogramming process. We found that several transcriptional waves that appeared during the reprogramming process may play a crucial role at different stages of mammary lineage conversion. It started with strong suppression on the TGF-β signaling pathway, forcing the fibroblast out of the cell cycle and overcoming multiple reprogramming barriers. Subsequently, the activation of epithelial and hormonal signaling pathways leads to the expression of genes related to mammary lineage establishment and development. Therefore, our findings provide insights into the mechanism of chemical induction of non-mammary cells to acquire mammary lineage transformation *in vitro* and lay a foundation for the follow-up research on the development and differentiation of mammary epithelial cells and lactation.

## Results

### Transcriptome characteristics of the CiMEC reprogramming process induced by small-molecule compounds

**FIGURE 1:**
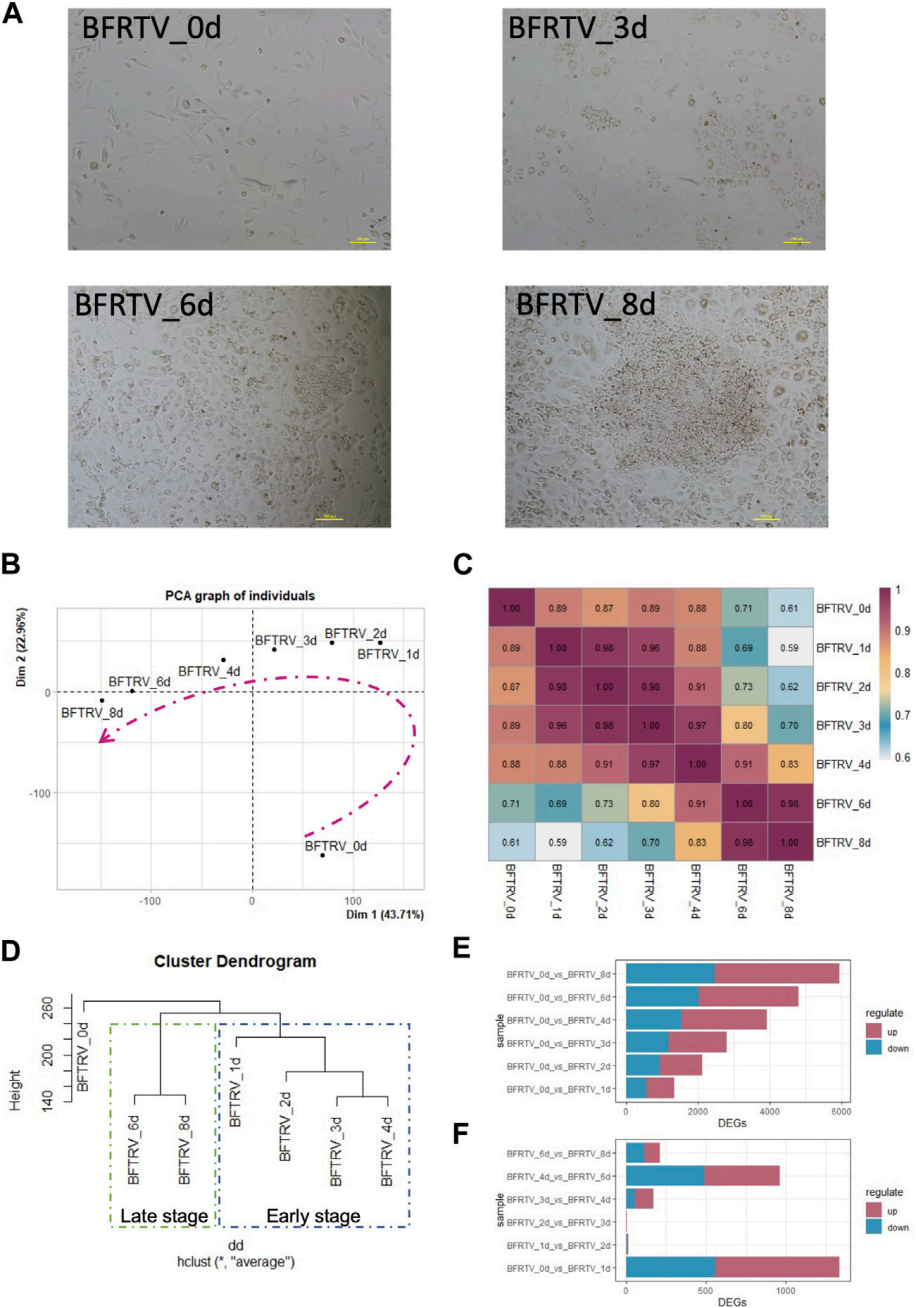
Gene expression changes in response to chemical induction during the reprogramming process. **(A)** Representative bright field images showing cell morphology change in induced cells during the reprogramming process (0, 3, 6, and 8 days) under BFRTV induction. Scale bars, 100 μm. **(B)** Principal component analysis for all samples. Dashed line indicates the conversion trajectory. It is important to note that BFRTV_0d samples were before BFRTV induction, and BFRTV_1d, BFRTV_2d, BFRTV_3d, BFRTV_4d, BFRTV_6d, and BFRTV_8d samples were post BFRTV induction. **(C)** Heatmap displaying correlations among different reprogramming time points according to the gene expression pattern. **(D)** Hierarchical clustering among all samples based on the expression of 29,107 genes. **(E)** Histogram showing the number of DEGs (*p* < 0.05, fold change >2) between BFRTV_1d, BFRTV_2d, BFRTV_3d, BFRTV_4d, BFRTV_6d, and BFRTV_8d samples, in all the pair-wise comparisons, and BFRTV_0d. **(F)** Histogram of the number of DEGs in pair-wise comparisons among adjacent time points.

Previously, we established that effective conversion of fibroblasts into functional CiMECs could be achieved by inducing a cocktail of five small-molecule compounds (BFRTV). After induction, the fibroblasts started to form epithelial-like cells, which eventually resulted in the formation of large, compact epithelial-like colonies with strong refractive edges by day 8 (Figure 1A). To further understand the molecular mechanisms underlying this reprogramming process, we collected samples from seven time points individually (0, 1, 2, 3, 4, 6, and 8 days post induction) and investigated the transcriptome changes during the chemical treatment using mRNA-sequencing technology (bulk mRNA-seq). When comparing the global gene expression pattern among different samples, the principal component analysis (PCA) (Figure 1B), correlation analysis, and hierarchical clustering showed a clear shift of the overall gene profile before and after BFRTV induction (Figure 1C). The PCA also showed that these samples (days 1–8) that were closer in time were mapped closer on the plot, indicating that BFRTV induction steadily pushed fibroblast through a conversion trajectory. Moreover, the hierarchical clustering analysis showed that cells clustered according to time changes and the reprogramming process could be divided into two stages: the early stage (days 0–4) and the late stage (days 6–8) (Figure 1D).

Then, we performed pair-wise differential expression analysis for samples from different time points as mentioned previously (fold change >2, adjusted *p* < 0.05). The results showed that compared to the day 0 samples, the number of upregulated and downregulated differentially expressed genes (DEGs) showed a steady increase among the treatments (Figure 1E). However, when samples from adjacent time points were compared (Figure 1F), the most dramatic change occurred within the first day of treatment. In conclusion, although the transcription level of reprogrammed cells has been changing steadily after induction, the most drastic change occurred on the first day.

**FIGURE 2:**
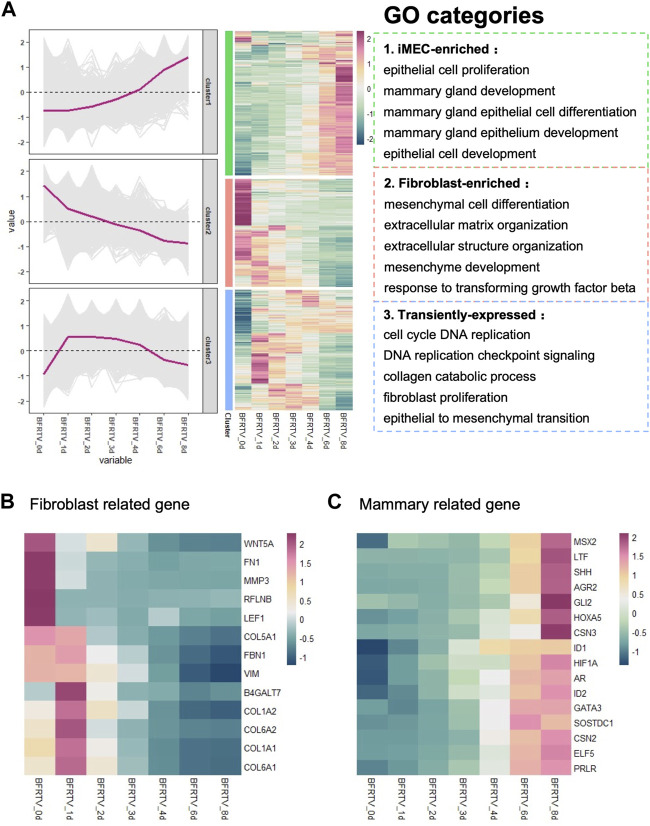
GO analysis of bulk RNA-seq data showed transition from fibroblasts to mammary epithelial cells. **(A)** Hierarchical clustering and heatmap of RNA-seq data showing all the differentially expressed genes (6,995 DEGs) among all samples. Red color indicates the high expression level, whereas green color indicates the low expression level. DEGs were divided into three clusters based on their expression patterns. Gene expression trend line is present in the middle. GO categories associated with each cluster are shown on the right. **(B)** Heatmap showing the expression pattern of fibroblast-related genes. **(C)** Heatmap showed the expression pattern of mammary development-associated genes.

After analyzing the overall changes in the DEG profile, we looked into the detailed categories of the DEGs during chemical reprogramming. Using a hierarchical clustering method, all the samples have a total of 8,726 DEGs that could be classified into three clusters based on their expression patterns: the first cluster of genes (green box) that were upregulated during the chemical reprogramming can be enriched into Gene Ontology (GO) terms related to mammary development, which means the mammary-related program was activated gradually. In addition, the second cluster of genes (red box), with rapid downregulation, has the GO terms related to response to transforming growth factor beta and mesenchymal-related terms, which indicated the downregulation of the somatic cell expression pattern. The third cluster of genes (blue box) showed a transient disturbance, which involved biological processes including the cell cycle and epithelial-to-mesenchymal transition, and it indicates that there is a transitional intermediate transcription wave in the early stage before activating the mammary gland signal (Figure 2A).

We further investigated the cell identity marker genes among the DEGs and analyzed their pattern of changes during the reprogramming process. The results showed that the expression of fibroblast characteristic genes was gradually inhibited (Figure 2B), followed by the activation of prolactin-related genes *PRLR* and *ELF5*, the mammary gland development genes *GATA3*, *GLI2*, *IDs*, *HOXA5*, and *SHH*, and the mammary bud gene *SOSTDC1*, which began to show upregulation at day 3. Importantly, the milk protein genes *CSN2*, *CSN3*, *LTF*, and *AGR2* were significantly upregulated at the late stage (Figure 2C). Therefore, these results indicate that small-molecule compounds significantly suppress fibroblast characteristics in the early stage, while the mammary development genes progressively activated the expression during reprogramming and finally acquired the mammary program at the late stage.

### Fibroblasts rapidly respond to chemical induction on the first day of reprogramming

**FIGURE 3:**
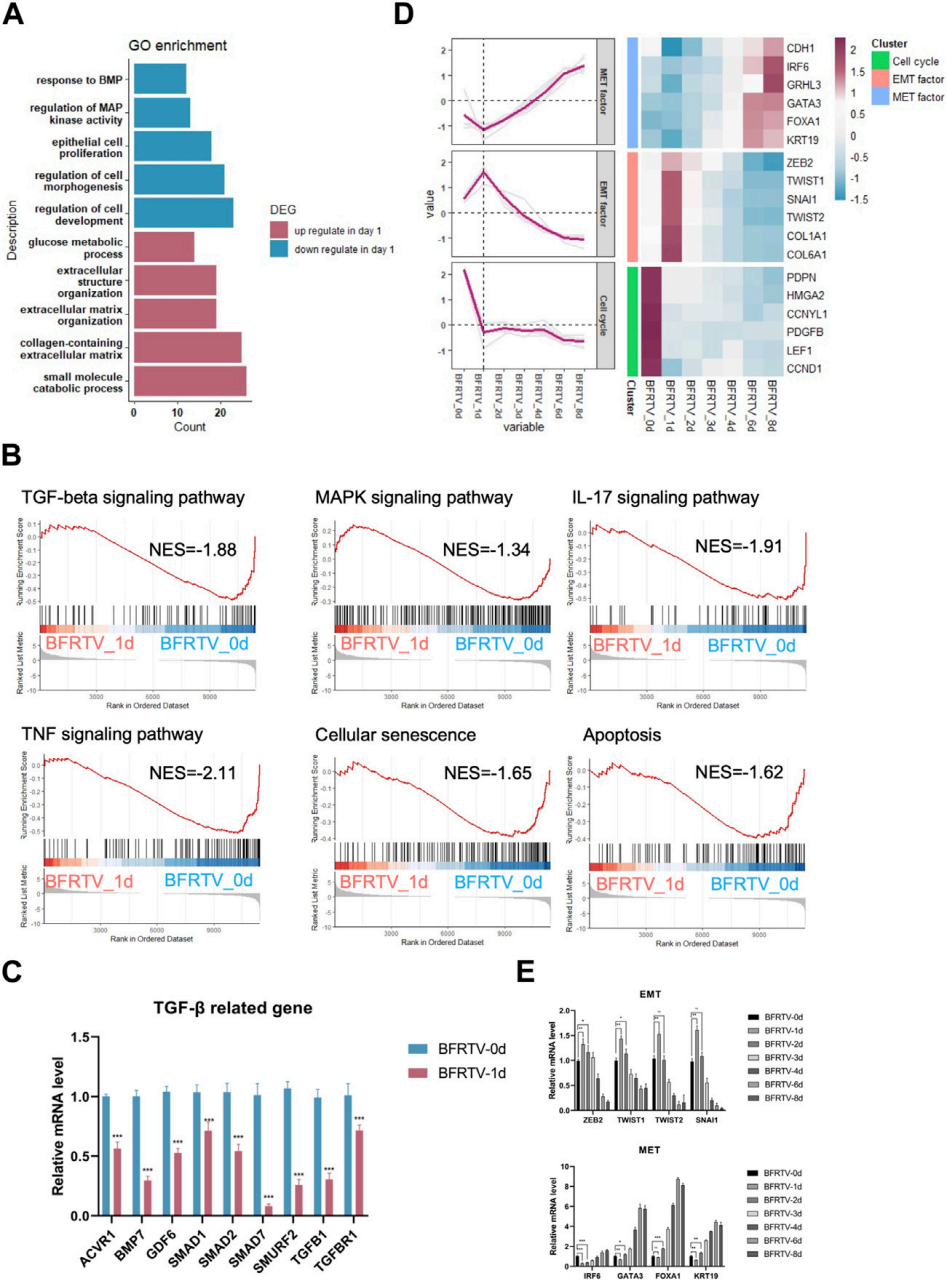
Bulk RNA-seq analyses data showed early genetic alternations of signaling pathways in response to chemically induction. **(A)** GO terms associated with upregulated and downregulated DEGs on the day 1 sample. **(B)** GSEA of day 1 samples compared to the day 0 sample revealed the early genetic alternations of signaling pathways in response to induction, and the TGF-beta signaling pathway and the reprogramming barrier signaling pathways were suppressed on day 1. For all KEGG terms, *p*-value is <0.05. **(C)** qPCR was used to detect changes in the expression of TGF-β-related genes (*ACVR1*, *BMP7*, *GDF6*, *SMAD1*, *SMAD2*, *SMURF2*, *TGFB1*, and *TGFBR1*). Compared to those of BFRTV-0d cells, the expression levels of TGF marker genes were significantly downregulated in BFRTV-1d cells. *p < 0.05, **p < 0.01, and ***p < 0.001. **(D)** Line graph (left) of EMT–MET factors and cell cycle gene expression pattern corresponding to the heatmap (right). The sequential EMT–MET process and the downregulation of cell cycle genes indicated the initiation of reprogramming. **(E)** qPCR was used to detect changes in the expression of EMT–MET-related genes. EMT-related genes (*ZEB2*, *TWIST1*, *TWIST2*, and *SNAI1*) showed a transient upregulation in BFRTV-1d cells, and the MET-related genes (*IRF6*, *GATA3*, *FOXA1*, and *KRT19*) upregulated from BFRTV-2d.

In order to further explore the molecular events occurring during reprogramming, we first analyzed the characteristics on the first day after induction. Compared to the initial day 0 sample, there were 779 upregulated and 557 downregulated DEGs on day 1 (Figure 1F). The GO analysis showed that genes related to extracellular morphology were activated, while genes related to cell development and differentiation were suppressed after induction (Figure 3A). The gene set enrichment analysis (GSEA) on day 1 samples revealed a significant downregulation of TGF-β and MAPK signaling pathways. Additionally, cellular senescence and apoptosis signaling pathways, together with the pro-inflammation pathways including TNF and IL17, were significantly enriched on day 1 downregulated genes (Figure 3B). Following the enrichment analysis, we investigated some individual genes and analyzed their expression pattern. Although most fibroblast marker genes began to show a downward trend after induction, such as TGF-β signaling-related genes were downregulated, EMT factors showed an upregulation from day 0 to day 1 and continued downregulation after day 1, while MET factors showed the opposite expression pattern (Figure 2B; Figure 3C, D), and the changes in the expression of TGF-β signaling-related genes and EMT–MET factors were also confirmed by qPCR (Figure 3C, E). At the same time, cell cycle genes were also downregulated on day 1 (Figure 3D), which means the cells entered the reprogramming state. Therefore, these findings indicate that the reprogrammed cells simultaneously inhibited pro-inflammatory, senescence, and apoptosis-related pathways on day 1, which may ensure the survival of cells and prepare them for continuing to enter the next state.

### Mammary lineage specific during the intermediate state

**FIGURE 4:**
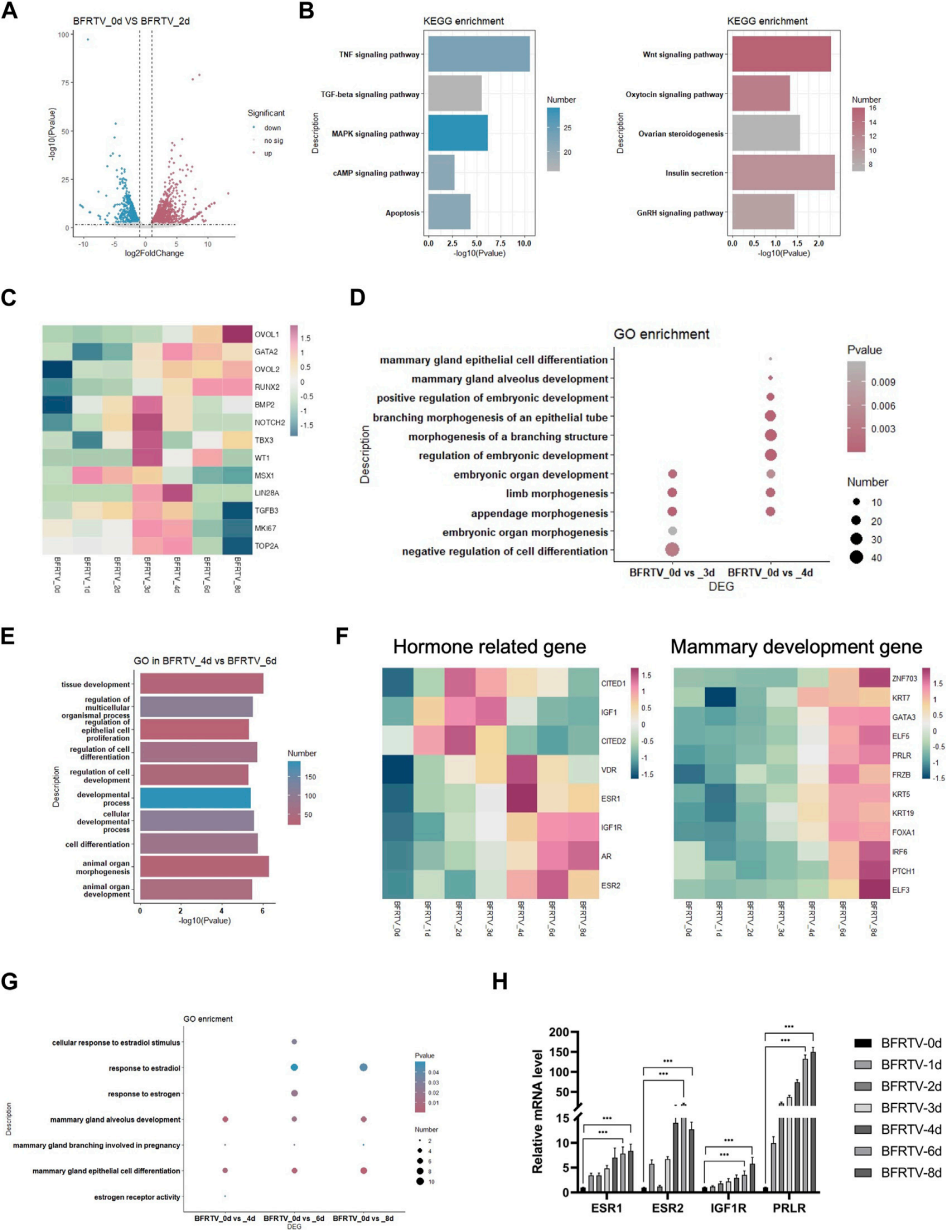
Bulk RNA-seq analyses data showed the reprogramming cells enter an intermediated plastic stage and rapidly acquire mammary lineage. **(A)** Volcano plot showing the DEGs on the day 2 sample. **(B)** KEGG terms associated with upregulated and downregulated DEGs on the day 2 sample, which correspond to the color in **(A)**. **(C)** Heatmap showing the expression pattern of intermediate plastic state-related genes and mammary epithelial-related genes. **(D)** Dot plot showing the GO terms related to the intermediate plastic state. **(E)** GO terms associated with DEGs on day 4 vs. day 6. **(F)** Heatmap showing the expression pattern of hormone-related (left) and mammary development (right) genes. **(G)** Dot plot showing the GO terms related to mammary lineage differentiation. **(H)** qPCR was used to detect changes in the expression of hormone-related genes (*ESR1*, *ESR2*, *IGF1R*, and *PRLR*). *p < 0.05, **p < 0.01, and ***p < 0.001.

In the subsequent early stage of the reprogramming process, with the epithelization of the cells, the day 2 sample showed a persistent suppression of TGF, MAPK, and reprogram barrier signals, and there was activation observed in the development-related signals, such as the WNT and insulin signaling pathways (Figure 4A, B). The following day 3 and day 4 samples showed an intermediate plastic state. We observed the upregulation of a panel of genes that were involved in early embryonic development (*NOTCH2* and *BMP2*) and cell proliferation (*TOP2A* and *MKI67*), and the plastic-identified genes such as *LIN28A* and *MSX1* also showed a transient activation (Figure 4C). GO analysis showed an upregulation of embryonic developmental features with limb development. Different from the day 3 sample that has the term “negative regulation of cell differentiation,” the day 4 sample activated the mammary differentiation signal that was enriched in the terms of “mammary epithelial cell differentiation” and “mammary alveolus development” (Figure 4D). Meanwhile, the mammary cell fate regulators *OVOL2* and *RUNX2* were gradually upregulated during this state (Figure 4C). These findings suggest that, after the sequential EMT-MET, the reprogramming cells go through a brief intermediate plastic state and rapidly acquire the mammary lineage.

In the adjacent time point sample DEG analysis, we found that there were up to 965 DEGs that were identified during the early-to-late-stage conversion (day 4–day 6), and GO enrichment showed that these genes were enriched in the terms such as “cell differentiation” and “animal organ development” (Figure 4E). Similar to the mammary development process *in vivo*, the expression of hormone-related genes (*ESRs*, *VDR*, and *AR*) was upregulated before the mammary development genes (Figure 4F). In addition, the qPCR revealed a similar pattern of hormone gene expression (Figure 4H). In addition, the GO analysis of DEGs in comparison with the day 0 sample showed that estrogen and mammary development-related terms were enriched in the reprogramming cells that have acquired the mammary lineage (Figure 4G), indicating that the reprogrammed cells underwent differentiation under the mammary lineage.

### scRNA-seq analyses reveal the reprogramming route was committed early

**FIGURE 5:**
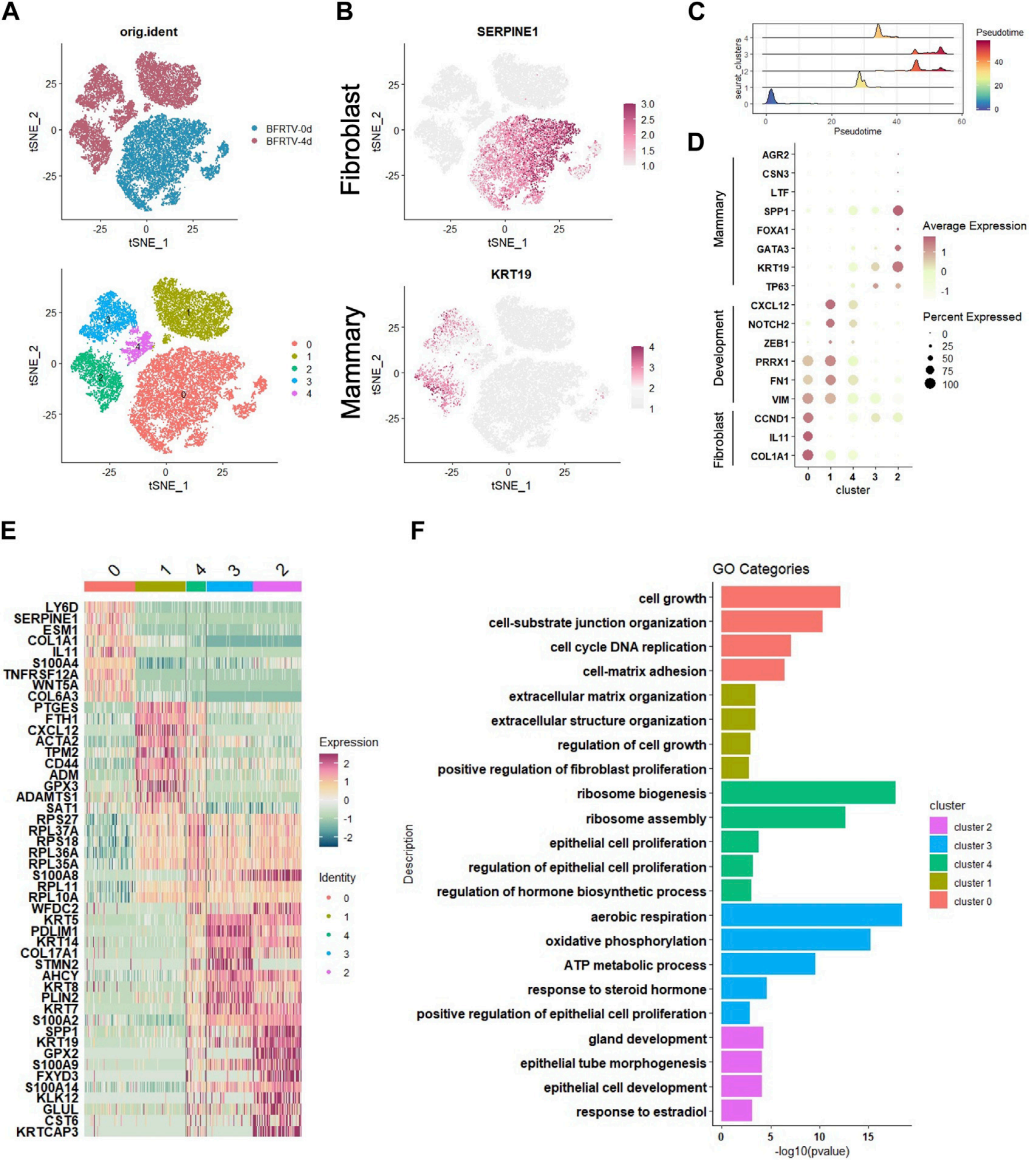
scRNA-seq analyses data showed the early genetic alternations in response to chemical induction. **(A)** tSNE visualization of the two samples of cells (up), BFRTV-0d and BFRTV-4d, that were divided into five clusters (down). **(B)** tSNE visualization of the fibroblast (up) and mammary (down) marker gene in scRNA-seq data of BFRTV-0d and BFRTV-4d. **(C)** Ridge plot showed the pseudo-time sort of each cluster from BFRTV-0d to BFRTV-4d. **(D)** Dot plot showing the marker genes in each cluster. **(E)** Heatmap showing the upregulated DEGs in each cluster. **(F)** GO analysis showing enriched terms in each indicated gene clusters.

To dissect the molecular events during the early stage of the reprogramming process, we further analyzed the scRNA-seq data collected on day 0 (BFRTV_0d) and day 4 (BFRTV_4d) samples. Using the unsupervised dimensional reduction and visualization method of *t*-SNE, we clustered cells into five clusters (Figure 5A). According to the marker genes for each cluster and the stages of the cells collected, we first identified the cells of cluster 0 that were marked with *SERPINE1* as starting fibroblasts (Figure 5B). The pseudo-time analysis showed that cluster 0 was followed by cluster 1, and cluster 4 connects cluster 1 and cluster 2/3. Then, we determined that the cells of cluster 1 maintained some fibroblast characteristics such as *FTH1* and *ACTA2*, and the EMT factors *VIM*, *PRRX1*, and *ZEB1* showed a transient upregulation, followed by immediate downregulation in cluster 4 (Figure 5C, D). The cells of cluster 2 and cluster 3 that were marked by *KRT19* had been successfully reprogrammed into induced mammary epithelial cells (Figure 5B); regarding the comparison between cluster 2 and cluster 3, the mammary lactation-related genes *SPP1*, *AGR2*, *CSN3*, and *LTF* were strongly expressed in the cells of cluster 2, which may indicate that the cells of cluster 2 represent more mature mammary epithelial cells (Figure 5C). GO analysis that enriched terms in each indicated gene cluster showed the mesenchymal characteristics in cluster 0 and cluster 1, while the rest of the clusters showed the epithelial characteristics. In addition, the hormone-related term and gland development term were enriched in clusters 2 and 3. Our scRNA data analysis showed that the reprogramming process first downregulated the fibroblast program and underwent the sequential EMT–MET, and the cells then epithelialized and acquired the mammary lineage rapidly, which was similar to the results of mRNA-seq analyses.

### Gene network analyses reveal the connection between TGF-β signal disturbance and the mammary development signal activation

**FIGURE 6:**
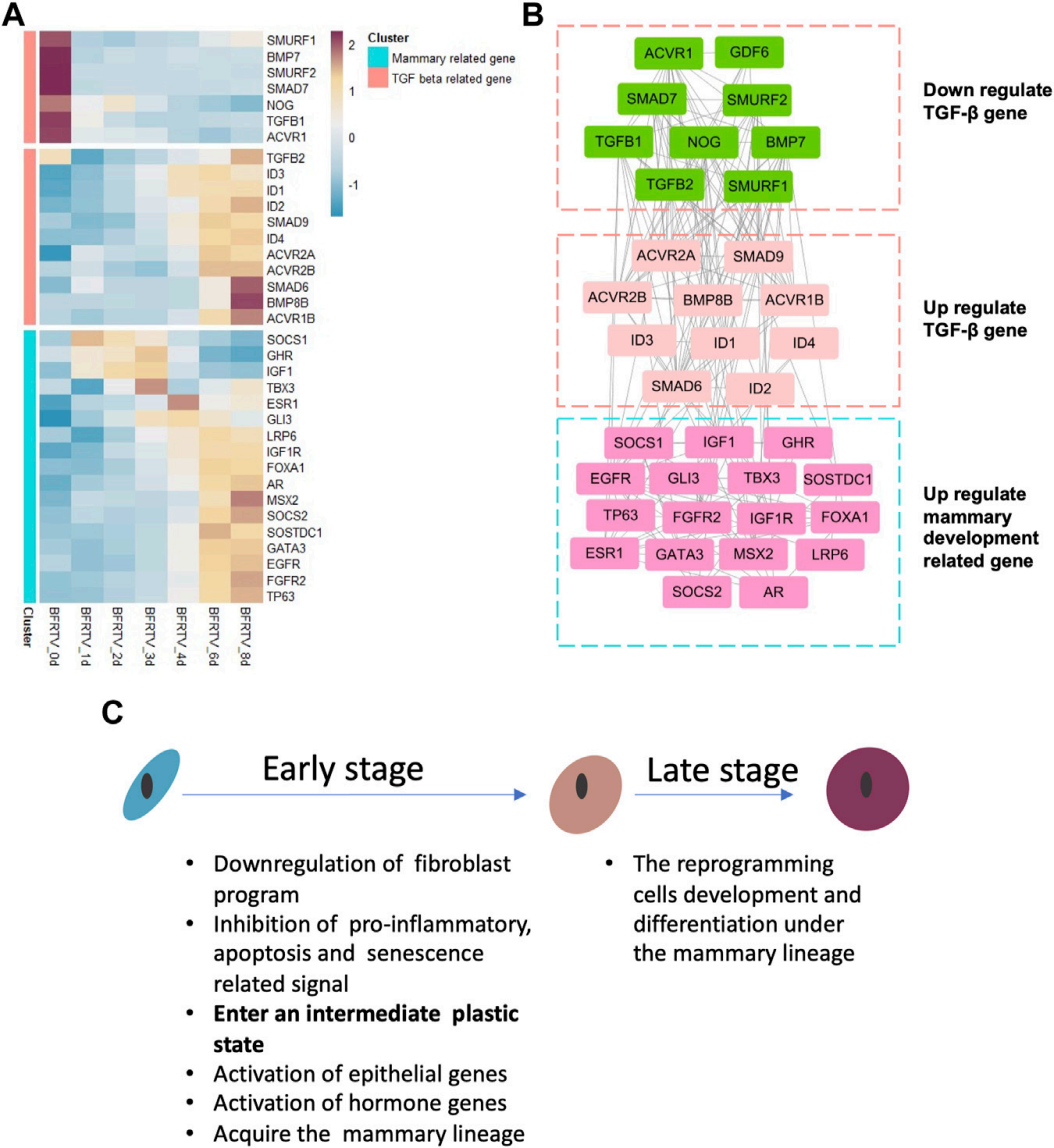
Gene co-expression network during chemical reprogramming. **(A)** TGF-β-related gene expression during the reprogramming process in bulk mRNA-seq. **(B)** Correlation network of genes shown in panel A. Different clusters had been grouped together in three squares. Color on the wireframe corresponds to the color bar in **(A)**. **(C)** Schematic diagram for key molecular events during the reprogramming process.

Our previous results identified RepSox as the major effector in small-molecule compound induction. In this study, we focused on the relationship between the TGF-β signaling pathway and the mammary signal during reprogramming. So, we characterized the genes on the TGF-β signaling pathway and found that not all the related genes have the same downward trend (Figure 6A). Our results showed that downregulated TGF-β genes were related to the maintenance of fibroblast characteristics. However, with the gradual epithelialization of reprogrammed cells, the ID family, which plays an important role in embryonic development and mammary gland development, is significantly upregulated. Moreover, we analyzed the correlation between these TGF signal-related genes and mammary development regulatory genes and found that the upregulated TGF-related genes were related to mammary development genes (Figure 6B). These findings suggest that TGF-β perturbations of signaling factors may play a crucial role in reprogramming cells that select mammary lineage.

## Discussion

In this work, we utilized two sequencing technologies to analyze the transcriptional level changes during the reprogramming of fibroblasts into mammary epithelial cells. Our findings indicate that there were two stages during small-molecule compound (BFRTV) induction (Figure 6C). In the early stage, the TGF-β signaling pathway was significantly altered in the initial fibroblasts, suppressing the mesenchymal characteristics, pro-inflammatory pathways, and cell death-related pathways, which provided conditions for reprogramming to occur and promoted cell survival. In the late stage, the reprogramming cells sequentially activated the hormone and mammary development signals and expressed milk protein-related genes. The gene network analysis revealed how the regulatory network involving mammary development genes is gradually activated. These genes synergistically regulate cell lineage changes in a spatiotemporal manner. Therefore, our study clearly delineates the molecular events and trajectory in the rapid conversion of somatic cells into CiMECs by small-molecule compound induction.

Although the reprogramming cells gradually change over time, the transcriptional level change that occurred on the first day of reprogramming was the most dramatic in the process, which indicates that fibroblasts’ response to the BFRTV induction occurred on the first day. This is consistent with studies of other types of reprogrammed cells (Ma et al., 2019; Masserdotti et al., 2015; Wapinski et al., 2013). In the early stage of reprogramming, the cells usually need to overcome multiple reprogramming barriers at the same time to enter reprogramming; several studies have revealed that pro-inflammatory pathways, cellular senescence, and cell death may act as a roadblock during the reprogramming process (Haridhasapavalan et al., 2020). The pro-inflammatory pathway is fatal to the plasticity of reprogrammed cells (Guan et al., 2022), and this activation is often related to apoptosis, proliferation, and stress signals. In addition, the apoptosis and senescence usually act as the major limitation in reprogramming efficiency around the time of fate conversion (Phanthong et al., 2013). Whether these barrier signals can be effectively suppressed determines cells either successfully convert into reprogramming process or succumb to cell death. In addition, the sequential EMT–MET plays an indispensable role in the initiation of the reprogramming process. It is also a key point affecting the reprogramming efficiency (Li et al., 2010). The function of temporary EMT before MET at the early stage is to prepare cells for subsequent conversion that facilitated the reprogramming process (He et al., 2017; Liu et al., 2013). It is speculated that during this period, the bulk of reset of the epigenome occurs and that it could avoid being side-tracked to various dead ends of reprogramming (Gaeta et al., 2013). Our data showed that on the first day of reprogramming, the downregulation of pro-inflammatory, senescence, and apoptosis pathway-related genes, as well as the transient sequential EMT–MET process, occurred at the early stage of reprogramming. Based on this, we speculate that the change in these signals may be a key point for cells to undertake all necessary measures to maintain survival and prepare cells for further conversion.

An important aspect of our findings is that small molecules can rapidly convert fibroblast identity to mammary lineage through an intermediate plastic state without activating the pluripotent network. It is known that somatic cell reprogramming is a multistep process (Samavarchi-Tehrani et al., 2010). After overcoming the original lineage program, the reprogrammed cells enter an intermediate plastic state, the original somatic program is silenced, and the genes related to development are upregulated. In here, we found such an intermediate plastic state in the cells of day 3–day 4. Most of the upregulated genes are concentrated in embryonic developmental features with limb development, as well as the increased cell proliferation genes, indicating that the genes controlling development were likely to be reactivated in the reprogrammed cells, leading to a cell state similar to the early embryonic development state which is essential for reprogramming cells to acquire a new lineage. Although a temporary hybrid epithelial/mesenchymal phenotype (Bocci et al., 2019) and plastic-identified genes such as *LIN28A* and *MSX1* were activated during the intermediate state, due to the rapid activation of the mammary development signal, especially the mammary epithelial differentiation transcriptional gatekeeper *OVOL2* and mammary epithelial cell fate regulator *RUNX2* were activated at the same time (Owens et al., 2014; Watanabe et al., 2014), and the reprogramming cells rapidly entered into the mammary lineage. So, we identified our intermediate state as a lineage-limited plastic state. It is worth mentioning that the unstable hybrid E/M phenotype (Brown et al., 2022), rather than the EMT or plastic marker continuous strong activation, determines that the reprogramming cells do not have stem-like characteristics but quickly activate mammary lineage features, which is different from the pluripotent stem cells or other direct lineage reprogramming (Guan et al., 2022; He et al., 2017; Ishay-Ronen et al., 2019). We observe that no pluripotency and progenitor characteristics were present during the whole process, indicating that this reprogramming process does not undergo pluripotency and avoids the potential tumorigenic risk of reprogrammed cells in subsequent applications.

The hormones play an important role in the development and differentiation of mammary and the synthesis and regulation of milk proteins. Estrogen and prolactin-related genes, including *ESR1* (Gregorio et al., 2021), *CITED1* (Howlin et al., 2006), and *PRLR* (Gallego et al., 2001), were significantly upregulated on the third or fourth day after reprogramming. Moreover, the upregulation of hormone-related genes preceded that of mammary development-related genes. In particular, most of the mammary alveolar development genes and milk protein synthesis genes showed significant upregulation at the later stage of reprogramming (days 6–8), which is similar to the development process in the mammary gland. This indicated that the reprogrammed cells undergo a process of transdifferentiation into mammary epithelial cells (Slepicka et al., 2021). It is worth mentioning that no pluripotency and progenitor characteristics were observed during the whole process, which indicates that this reprogramming process does not undergo pluripotency and avoids the potential tumorigenic risk of reprogrammed cells in future applications.

In previous studies (Zhang et al., 2021), we have clearly identified that RepSox is critical to the occurrence of CiMEC reprogramming and the lineage determination of reprogrammed cells. RepSox can widely inhibit the TGF-β expression of signal pathway-related genes. It was found that the TGF-β signaling pathway-related genes have different expression patterns in different cell states. After reprogramming occurs, TGF-β genes that maintain fibroblast characteristics are downregulated. However, the BMP family gene that regulated embryonic development (Sozen et al., 2021) and ID family genes related to mammary gland development (de Candia et al., 2004) are significantly upregulated after cells undergo the intermediate state. The upregulation of these genes may play a role in the final selection of mammary lineage by reprogrammed cells.

## Conclusion

In conclusion, our transcriptome analysis reveals the molecular cascade induced by the reprogrammed cells in response to the small-molecule compound cocktail induction and delineated the route of the reprogrammed cell fate conversion process. The insights obtained from this study will help us to further improve the chemically induction system for obtaining mammary epithelial cells with milk secretory functions *in vitro* and, moreover, further investigate the regulatory mechanism of mammary epithelial cell fate decision.

## Materials and methods

### iMEC reprogramming

GEFs were induced by the BFRTV induction medium, which consists of the Neurobasal Medium (GIBCO), KnockOut DMEM-F12 (GIBCO), KSR (GIBCO), 100× N2 (GIBCO), 50× B27 (GIBCO) supplements, 1% Gluta-MAX (GIBCO), and supplemented with five small-molecule cocktails, 1 μM TTNPB (B),10 μM Forskolin (F), 10 μM RepSox (R), 10 μM tranylcypromine (T), and 500 μg/mL VPA (V). The culture was continued for 8 days, and the induction medium was refreshed every 2 days. More details are provided in the work of Zhang et al. (2021).

### Quantitative real-time polymerase chain reaction

Total RNA was extracted using the TRIzol reagent. The cDNA was synthesized by reverse transcription. The qPCR system comprised 2 × SYBR Green Mix (10 μL), primer mix (1 μL), template (1 μL), and ddH_2_O (8 μL). The qPCR parameters were as follows: 95°C for 30 s followed by 40 cycles of 95°C for 30 s and 60°C for 30 s. The GAPDH gene was used as a reference gene. The relative mRNA expression level of each gene from triplicate experiments was calculated using the 2^−ΔΔCT^ method. The primer pairs used are shown in Table 1.

**TABLE 1:** RT-PCR primer sequence.

Gene name	Sequence	5′-3′
GAPDH	Forward	GGA​AGC​TCG​TCA​TCA​ATG​GA
	Reverse	GCT​GAC​AAT​CTT​GAG​GGT​GT
ACVR1	Forward	GGC​CTA​ACA​TAC​CCA​ACA​GA
	Reverse	GAA​AGC​AAT​CAT​CAC​GAG​CAC
BMP7	Forward	ACT​CAG​CTC​AAT​GCC​ATC​TCC​G
	Reverse	CCA​GAG​CGC​CAC​GAA​GCT​GT
GDF6	Forward	ACG​CCA​TCA​TCC​AGA​CGC​TGA
	Reverse	GGA​AAC​CCG​GCA​GAT​CCC​ACA
SMAD1	Forward	GTA​TGC​CGA​ATG​CCT​CAG​TG
	Reverse	AAA​GCT​CAT​GCG​GAT​AGT​GC
SMAD2	Forward	GCA​ATC​TTT​GTG​CAG​AGC​CC
	Reverse	TGC​TTG​TTA​CCG​TCT​GCC​TT
SMAD7	Forward	GGC​TGT​GTT​GCT​GTG​AAT​CT
	Reverse	GCC​GAT​TTT​GCT​CCG​TAC​TT
SMURF2	Forward	ACC​TGC​TTC​AAT​CGA​ATA​GAC​A
	Reverse	TTG​CCC​AGA​TCC​ATC​AAC​CAC
TGFB1	Forward	ACA​CGC​AGT​ACA​GCA​AGG​TC
	Reverse	AGG​CGT​CAG​CAT​TAG​TAG​CCA​CA
TGFBR1	Forward	TAG​GCT​TAC​AGC​TTT​GCG​GAT
	Reverse	CAG​AAA​CAC​TGT​AAT​GCC​GTT​G
IRF6	Forward	TGA​AGC​AGC​TGT​ACC​GCA​TCC
	Reverse	ACA​GGC​CAC​TAT​CCA​CTT​GGG
GATA3	Forward	ACA​TCC​ACC​TGG​TTG​AAC​TTC​TCT​ACT​A
	Reverse	CTG​GTT​TTC​AGG​GCC​TTC​AG
FOXA1	Forward	GCA​TTG​CCC​GCC​AGC​TTG​CC
	Reverse	AGT​CGC​TGC​TCT​CGT​GCC​CTT
KRT19	Forward	CTC​CGG​GCA​TCG​ACC​TAG​CCA​A
	Reverse	CTC​CTT​GTT​CAG​CTC​CTC​GGT​CT
ZEB2	Forward	ATC​CCG​AAA​CGA​TAC​GAG​ATG
	Reverse	CAC​GCA​GGC​TCG​ATA​TCT​TC
TWIST1	Forward	GCT​CAG​CTA​CGC​CTT​CTC​GGT​CT
	Reverse	CTC​GCT​GTT​GCT​CAG​GCT​GTC​GT
TWIST2	Forward	CAG​CTA​CGC​CTT​CTC​CGT​GTG
	Reverse	CTT​CTT​GCT​GTA​GCG​CCG​TTT
SNAI1	Forward	AAC​CTT​CTC​CCG​AAT​GTC​CCT
	Reverse	TGC​AGC​TCA​CTG​TAA​TTG​GGT​CT
ESR1	Forward	TTG​CTG​GCT​ACA​TCG​TCT​CGG​TTC​CGT
	Reverse	AGG​CAT​GGT​GGA​GAT​CTT​TGA​C
ESR2	Forward	CGA​CTG​CGG​AAG​TGC​TAT​GAG
	Reverse	TCA​CTG​AGC​CTG​GGG​TTT​CTG
IGF1R	Forward	TGA​AGC​GCC​TGG​AGA​ACT​G
	Reverse	TCCTCGGCCTTGGAAATG
PRLR	Forward	ATA​TTC​AGC​AAG​GAG​CAA​GA
	Reverse	AAT​GAG​GAT​GGA​AGT​CAG​AG

### Bulk RNA-seq bioinformatic analysis

The bulk RNA-seq data were acquired from our previously published sequencing data. The data were analyzed on the Majorbio Cloud Platform (https://cloud.majorbio.com/); GO and KEGG analyses were performed using the functions enrichGO and gseaKEGG in the R package clusterProfiler (Wu et al., 2021). The correlation network was calculated by STRING (https://cn.string-db.org/) and visualized using Cytoscape (v3.9.0).

### ScRNA-seq bioinformatic analysis

The RDS files for sample BFRTV-0d and sample BFTRV-4d were obtained from Gene Expression Omnibus (GEO) (#GSE142551). The expression matrices were loaded into R v.4.1.0 (Giorgi et al., 2022). Cell-level quality control was performed by filtering the cells by mitochondrial gene percentages less than 0.8. The expression level of each gene in each cell was normalized using the function NormalizeData with the default parameters. Cluster-level quality control was performed after the standard Seurat clustering pipeline was implemented using the following functions in order: FindVariableFeatures with all features, ScaleData, RunPCA, FindNeighbors with the first 16 principal components (PCs), and FindClusters with resolution 0.1, otherwise default settings. Markers were calculated using the function FindMarkers in Seurat (Hao et al., 2021), which compared each cluster to all of the other clusters to form a dynamic gene pool. To annotate the function of gene groups showing different expression patterns, we performed GO analysis of all gene groups using the function enrichGO in the R package clusterProfiler. We used typical GO terms of each group for visualization.

### Statistical analysis

All data were statistically analyzed using Prism 8.0 (GraphPad Software Inc., United States). Data were expressed as mean ± standard error. Differences among multiple comparisons were performed using two-way analysis of variance (ANOVA). Differences between two groups were analyzed by Student’s *t* test (two-tailed) based on normal distribution. Correlation analysis was performed using the Spearman method. *p* < 0.05 was considered statistically significant.

## Data Availability

Publicly available datasets were analyzed in this study. This data can be found here: https://www.ncbi.nlm.nih.gov/geo/; GSE14255.

## Abbreviations

CiMECs, chemically induced mammary epithelial cells; GEFs, goat ear fibroblasts.
